# Vitamin D Supplementation Should Be Considered for the Treatment of COVID-19 Infection in Malaysia in View of the High Prevalence of Vitamin D Deficiency

**DOI:** 10.21315/mjms2021.28.6.15

**Published:** 2021-12-22

**Authors:** Shamin MOHD SAFFIAN

**Affiliations:** Centre for Quality Management of Medicines, Faculty of Pharmacy, Universiti Kebangsaan Malaysia, Kuala Lumpur, Malaysia

Dear Editor,

The significance of vitamin D requirements for bone and muscle health is well recognised and has been repeatedly discussed in the literature. However, many readers might not be aware of the role of vitamin D in the immune system and the growing evidence that vitamin D plays a crucial role in COVID-19 infection (1, 2). This letter briefly describes observational and intervention studies on the relationship between vitamin D and COVID-19 infection and provides some insights into the local situation in Malaysia.

Recently, Pereira et al. (3) conducted a systematic review of 27 observational studies that investigated the relationship between vitamin D deficiency and the severity of COVID-19 infection. The 27 studies were conducted worldwide, including Europe (*n* = 15), the Mediterranean (*n* = 2), the USA (*n* = 3), the Western Pacific (*n* = 3), South Asia (*n* = 2) and Indonesia (*n* = 2). They found that patients with severe COVID-19 had markedly low vitamin D levels and the fatality rate was high in vitamin D-deficient groups, but vitamin D deficiency was not associated with a higher risk of COVID-19 infection. It is important to note that the geographical distribution was important because COVID-19 fatalities were found to be highly correlated to latitude, which is linked to vitamin D deficiency (4). While not necessarily causative, because respiratory tract infections can lower vitamin D levels (5), these studies point towards a strong relationship between vitamin D levels and the severity of COVID-19 infection.

Several randomised controlled trials (RCTs) have been conducted to investigate whether vitamin D supplementation can improve clinical outcomes in COVID-19 infections. A group of Spanish researchers treated 50 randomised patients with high doses of calcifediol plus standard care, while 26 others received only standard care. They showed that calcifediol-treated patients with COVID-19 infection required significantly less intensive care unit (ICU) treatment in hospitalised patients (6). A clinical trial conducted in India supplemented 60,000 IU of cholecalciferol in 16 COVID-19 patients, while 20 other patients received a placebo. All the patients in the trial were vitamin D deficient (defined as 25(OH)D level < 20 ng/mL). After administering the vitamin D supplementation for a week, the investigators found that it improved various COVID-19 severity markers (7). Also, two French studies (8, 9) that used a quasi-experimental study design found that vitamin D supplementation improved survival.

However, not all trials have shown improvement in COVID-19-infected patients with vitamin D supplementation. A Brazilian RCT (10) published in *JAMA* did not demonstrate the same effect as the other trials mentioned here. However, as detailed by the editor of *JAMA* (11), several limitations would limit the generalisability of their findings.

One may next ask, how prevalent is vitamin D deficiency in Malaysia? A local longitudinal study called *MyHeARTs* found that 78.9% of 1361 sampled 13-year-old Malaysian adolescents had vitamin D deficiency (12, 13). Another study of 57 pregnant women sampled from the Kuala Lumpur area found that 91% were vitamin D deficient (14). This was echoed by another larger study, where 42.4% of 535 pregnant women were found to be vitamin D deficient (15). It has also been shown that urban women in Malaysia had significantly lower vitamin D levels compared to rural women (16), while a slightly dated but still relevant study showed that approximately 68% of Malay adults in Kuala Lumpur had insufficient vitamin D levels (*n* = 380) (17). Overarchingly, these studies have repeatedly indicated that many Malaysians do not have the recommended level of vitamin D.

It is well known that adequate exposure to sunlight together with a healthy diet is the best way to increase circulating vitamin D levels. However, because of our lifestyles in the new norm, this might not be a feasible option for everyone. Therefore, vitamin D supplementation for individuals who are deficient has been suggested by some authors as a method to increase circulating vitamin D and consequently improve innate and adaptive immunity (1). However, it should be noted that vitamin D supplementation should only be part of general measures in the fight against COVID-19. It does not, in any way, negate the need for other measures, such as a healthy diet, adequate sleep and physical exercise, avoiding and relieving stress and other proven preventative measures, including physical distancing, hand washing and wearing face masks.
